# Synergistic Antioxidant and Cytoprotective Effects of *Thunbergia laurifolia* Lindl and *Zingiber officinale* Extracts Against PM2.5-Induced Oxidative Stress in A549 and HepG2 Cells

**DOI:** 10.3390/foods14030517

**Published:** 2025-02-05

**Authors:** Chattip Sunthrarak, Kakanang Posridee, Parinya Noisa, Soon-Mi Shim, Siwatt Thaiudom, Anant Oonsivilai, Ratchadaporn Oonsivilai

**Affiliations:** 1 Health and Wellness Research Unit, School of Food Technology, Institute of Agricultural Technology, Suranaree University of Technology, Nakhon Ratchasima 30000, Thailand; chattip.ct@gmail.com (C.S.); posridee.ka@gmail.com (K.P.); thaiudom@sut.ac.th (S.T.); 2 School of Biotechnology, Institute of Agricultural Technology, Suranaree University of Technology, Nakhon Ratchasima 30000, Thailand; p.noisa@sut.ac.th; 3 Department of Food Science and Biotechnology, Sejong University, Seoul 05006, Republic of Korea; soonmishim@sejong.ac.kr; 4 School of Electrical Engineering, Institute of Engineering, Suranaree University of Technology, Nakhon Ratchasima 30000, Thailand

**Keywords:** particle matter, synergistic, gene expression, antioxidant, oxidative stress

## Abstract

PM2.5, a fine particulate matter, poses considerable health risks. When inhaled, PM2.5 can deeply penetrate the lungs, triggering respiratory issues such as pneumonia and bronchitis, aggravating heart and lung conditions, increasing the risk of lung cancer, causing cardiovascular problems, and affecting the nervous, immune, and reproductive systems. This study investigated the protective effects of the combination extract (CRGE) of *Thunbergia laurifolia* Lindl. (Rang Chuet) water extract (RWE), and *Zingiber officinale* (ginger) ethanol extract (GEE) against PM2.5-induced oxidative stress in A549 and HepG2 cells. CRGE exhibited superior cytoprotective effects compared to the single extracts (RWE and GEE) by significantly reducing PM2.5-induced cytotoxicity and reactive oxygen species production while enhancing antioxidant enzyme activity. To investigate the effects of PM2.5 exposure on cellular responses, gene expression analysis was conducted on a panel of antioxidant enzymes (heme oxygenase 1, superoxide dismutase, catalase, and glutathione peroxidase), the phase II detoxification enzyme NQO1, and the inflammatory markers interleukin (IL)-6 and IL-8 using the A549 and HepG2 cell lines. CRGE treatment effectively reversed the PM2.5-mediated changes in gene expression in both cell lines, suggesting that it may help restore cellular antioxidant defense mechanisms and mitigate PM2.5-induced oxidative stress. This study showed that CRGE holds promise as a natural antioxidant and cytoprotective agent against PM2.5-induced oxidative stress. Further studies are required to investigate the underlying mechanisms and confirm the efficacy of CRGE in vivo.

## 1. Introduction

Several chronic conditions, including aging, Alzheimer’s disease, atherosclerosis, diabetes, hypertension, kidney disease, and cancer, are adversely affected by oxidative stress [1]. Epidemiological studies have indicated that consumption of plant-based diets may help reduce oxidative damage [2]. Polyphenols are a class of phytochemical compounds that are specialized plant metabolites that benefit human health by counteracting the pathological effects of oxidative stress [3]. As a result, numerous studies have attempted to assess the health benefits of antioxidant activity [4,5,6].

*Thunbergia laurifolia* Lindl. belongs to the *Acanthaceae* family and is found throughout India and Southeast Asia. It is referred to as Rang Chuet (RC) in Thai and is used in traditional Thai medicine. Leaves of RC are widely used as an antidote for poisons. This plant exhibits antioxidant, anti-inflammatory, antimicrobial, anti-diabetic, antipyretic, and anti-hyaluronidase activities, and significant research interest in its bioactive and unique phytochemical compounds has been shown [7,8,9,10,11]. RC has a rich history in traditional Thai medicine as an antidote that counteracts the effects of poisons and venoms. It interferes with the ability of the poison to cause harm to the body. In addition, RC has anti-inflammatory and antipyretic properties [8]. Modern science has revealed extensive quantities of phytochemicals, such as flavonoids (apigenin, luteolin) and steroids (Beta-sitosterol, stigmasterol, alpha-sinasterol), within RC. Aqueous extracts protect against various poisonings, whereas the dried roots are used to treat inflammation and fever. RC leaf extracts protect the liver from ethanol damage and may even have chemo-preventive applications [10]. This multifunctional medicinal plant, supported by traditional and scientific exploration, requires further investigation to fully uncover its full therapeutic potential [10].

*Z. officinale* Roscoe (ginger) is well known for its numerous health benefits. Revered for centuries for its culinary and medicinal properties, ginger has recently gained significant attention for its potent antioxidant activity, attributed to a diverse array of phytochemicals [12,13]. Gingerol and zingerone, the primary active compounds, exert antioxidant effects by reducing the free radical scavenging activity [14]. Furthermore, ginger has anti-inflammatory properties owing to the combination of 1-dehydro-[6]-gingerdione, 6-shogaol, 6-dehydroshogaol, and hexahydrocurcumin [15]. Ginger water contains significant bioactive compounds, including phenolic gems such as gingerols, shogaols, and paradols alongside flavorful terpenoids such as zingiberene and α-/β-bisabolene [16,17]. Antioxidant flavonoids, such as quercetin and kaempferol, enhance their value as a resource, whereas saponins, such as zingiberosides offer potential anticancer and immune-modulatory benefits [16,18]. The phytochemical profile of ginger exhibits significant variability depending on the cultivar selection, cultivation conditions, extraction methods, and storage practices. This inherent variety renders each batch composition distinct, and comprehensive characterization is required [12,17].

Owing to its culinary versatility, ginger water extract exhibits a robust antioxidant capacity, containing significant levels of neutralizing free radicals and reactive oxygen species (ROS) [13,18]. Both in vitro and in vivo studies have suggested a potential role of this substance in reducing oxidative stress, protecting cells from damage, and inhibiting lipid peroxidation. However, further research is required to confirm these preliminary findings [13]. The potency of the extract correlated with its phenolic content suggests a synergistic effect in promoting cellular defense [13]. Although ginger excels in neutralizing free radicals, its benefits are more extensive. Studies have suggested that it can regulate the gene expression of key antioxidant enzymes, such as superoxide dismutase (SOD), catalase (CAT), and glutathione peroxidase (GPx), which essentially increases the volume of their protective chorus within cells [19,20]. It orchestrates the Nrf2 signaling pathway, which is important in antioxidant defense [19]. Ginger can also reduce the harmful effects of inflammation. This is accomplished by blocking genes that trigger inflammation within the cells. This helps reduce internal activity within the cell [20]. When extracts from different plants are combined, beneficial compounds work together more effectively [21]. Although polyphenols provide numerous health benefits, their interactions within the body are complex. Antagonistic effects, such as binding or inhibiting the absorption of other polyphenols, can diminish their overall bioavailability and ultimately reduce their beneficial effects on health. These phytochemicals may possess similar structures, resulting in complementary effects on their antioxidant activities. The interactions of phytochemicals with food, plant extracts, and natural antioxidants have been reported in various in vitro and in vivo studies [22,23,24,25,26].

In addition to its culinary applications, ginger and its primary constituents, gingerols, and shogaols, possess substantially medicinal potential. A substantial body of preclinical research has demonstrated its efficacy in addressing a range of conditions, including diabetes, obesity, diarrhea, allergies, pain, fever, rheumatoid arthritis, inflammatory disorders, various cancers, and, more recently, coronavirus disease [27].

Oxidative stress, an imbalance between free radical production and antioxidant defense, can contribute to various health problems. Understanding the factors driving this imbalance and implementing dietary management strategies are crucial for maintaining well-being. Recently, well-being has gained prominence in health-related disciplines. This multifaceted construct is influenced by the interplay among social, economic, and health factors, including dietary patterns. Well-being encompasses a broad spectrum, incorporating objective factors, such as access to social support, sanitation infrastructure, employment opportunities, income levels, and educational attainment, alongside subjective assessments of individual well-being, commonly referred to as subjective well-being [28,29]. Studies have highlighted several contributors, including a diet rich in processed foods and deficient in essential nutrients, along with lifestyle factors, such as smoking, excessive alcohol consumption, and chronic stress [30]. Notably, dietary adjustments are powerful tools for combating oxidative stress. Increased consumption of antioxidant-rich fruits, vegetables, whole grains, nuts, and healthy fats, such as olive oil and fatty fish, can bolster defenses. In addition, limiting saturated and trans-fats, moderating caffeine and alcohol consumption, and addressing stress via healthy coping mechanisms can further support a balanced antioxidant system and promote overall health. Consulting a healthcare professional for personalized dietary guidance is always advisable [30].

According to the World Health Organization (WHO), 7 million deaths occur annually owing to exposure to air pollution. Surprisingly, >91% of the global population resides in areas where air pollution levels exceed WHO guidelines [31]. The particulate matter with a diameter of <2.5 µm (PM2.5) can penetrate deeply into the alveolar tissue of the respiratory tract, and inhaled PM2.5 can diffuse into the blood through microvasculature and then be transported throughout the body. Exposure to PM2.5 has been linked to various health problems, including respiratory diseases [31]. A significant portion of these fine particles is retained in the lungs, contributing to respiratory infections, systemic inflammation, and long-term health issues, such as cardiovascular disease, neurological disorders, and adverse effects on the liver, spleen, blood, skin, and reproductive system [31,32].

PM2.5, which comprises a complex mixture of heavy metals, transition metals, polycyclic aromatic hydrocarbons (PAHs), and other hazardous compounds, is influenced by diverse factors [32]. Exposure to PM2.5 can activate multiple harmful pathways, including oxidative damage, inflammation, cell autophagy, and apoptosis, as evidenced by previous studies. The principal mechanism by which PM2.5 induces cellular toxicity seems to be via increased oxidative stress, characterized by elevated levels of ROS and diminished SOD activity [32]. Multiple studies have reported inflammatory and oxidative stress–induced cytotoxicity mechanisms of PM2.5. Recent investigations of naturally derived polyphenols have shown potential as protective and preventive agents that influence a multitude of physiological processes via various signaling pathways. Dietary polyphenols may provide a promising therapeutic or preventive strategy to mitigate the health risks associated with air pollution, particularly from PM2.5 exposure [33]. For instance, pre-treating A549 cells with the antioxidant morin effectively protected against PM2.5-induced toxicity and restored gene expression levels [34].

Research has shown that RC has the potential to detoxify phase II enzymes. In addition, the intestinal transport of rosmarinic acid in RC extracts was mainly penetrated as conjugated forms with glucuronic acid and/or sulfate across Caco-2 cells and transported via passive diffusion [35]. However, the effects of RC on PM2.5-induced lung and liver cell models remain to be elucidated. The combination of bioactive constituents present in plant extracts and various natural products produces synergistic effects that enhance the activity of plant extracts [21]. Therefore, the synergistic antioxidant activity of the optimized mixture could be more effective at lowering the dose of plant extracts. RC and ginger extracts exhibited synergistic antioxidant activities. The synergistic effect of the combined extracts on cellular antioxidant activity was investigated in a previous study [27], which demonstrated synergism at certain ratios. To maximize their benefits, the impact of combined RC and ginger extracts in mitigating PM2.5, exposure requires further exploration. However, the antioxidative, oxidative stress reduction, and cytoprotective effects of Rang Chuet water extract (RWE), ginger ethanol extract (GEE), and their combination (CRGE) on particulate matter 2.5-(PM2.5)-exposed human lung epithelial (A549) cells and human hepatoma (HepG2) cell lines have not been investigated.

The present study investigated the cytoprotective potential of RC combined with ginger extracts against the adverse effects of PM2.5, in A549 and HepG2 cell models. This study will assess this by evaluating cellular antioxidant activities and analyzing changes in gene expression related to antioxidant, inflammatory, and detoxification pathways in cells exposed to PM2.5.

## 2. Materials and Methods

### 2.1. Chemicals and Reagents

1,1-diphenyl-2-picrylhydrazyl (DPPH) free radical, 2,4,6-tri (2-pyridyl)-striazine, ferric chloride, ferric chloride 6-hydrate, ferrous sulfate 7-hydrate, 2,2′-azino-bis (3-ethylbenzthiazoline-6-sulphonic acid), potassium persulfate, gallic acid, ascorbic acid, butylated hydroxytoluene, 6-hydroxy-2,5,7,8-tetramethylchroman-2-carboxylic acid, Folin–Ciocalteu phenol reagent, sodium acetate, anhydrous sodium carbonate, sodium nitrite, aluminum chloride, sodium hydroxide, and ferrozine were obtained from Sigma Aldrich Co. (St. Louis, MO, USA). Methanol, glacial acetic acid, and hydrochloric acid (37% *w*/*w*) were used for chemical analysis. All solvents, including 95% ethanol, were purchased from Mallinckrodt-Baker (Phillipsburg, NJ, USA).

### 2.2. PM2.5 Preparation

A Standard Reference Material (SRM 2786) sourced from the National Institute of Standards and Technology was used to represent fine PM2.5. This fine particulate matter was collected in Prague, Czech Republic, in 2005 and had an average particle diameter of approximately 2.8 µm. A certificate of analysis for the SRM 2786 used in this study is provided in the Supplementary Materials. To prepare a fresh stock suspension of SRM 2786, it was diluted in Dulbecco’s modified eagle medium (DMEM) to achieve a final concentration of 2000 µg/mL. This suspension was then sonicated for 5 min with a BANDELIN Electronic GmbH & Co., Berlin, Germany, and vortexed for 2 min prior to each experiment, following the protocol outlined by Xiong et al. [35].

### 2.3. Plant Preparation

A voucher specimen (Ratchadaporn 001) of RC leaves, collected in March 2021 from a Non-toxic Agriculture Cooperative under the Royal Initiative in Nakhon Ratchasima Province, Thailand, was deposited to the Herbarium of the School of Food Technology, Suranaree University of Technology. Fresh RC leaves were dehydrated to a moisture content of <13% using a microwave vacuum dryer. Dried RC was ground and sieved to a particle size of 0.2 mm. Commercial ginger powder was obtained from Now Foods (Bloomingdale, IL, USA). Both RC and ginger powders were stored at −20 °C until further use.

### 2.4. Extract Preparation

The various extracts were prepared as described by Oonsivilai et al. [7]. To prepare RWE, 100 mg of leaf powder was mixed with three 12-portion volumes of boiling water. The mixture was then incubated in a shaking water bath at 25 °C for 15 min. After each extraction, the mixture was centrifuged at 3000 g for 3 min. The combined filtrates were adjusted to a final volume of 50 mL and lyophilized for 48 h. The dried extract was stored at −20 °C until further use. For each combination, the leaf powders of the two plants were mixed in the following ratios of RWE to GEE: 1:10, 1:1, and 10:1. The total weight of each mixture was 100 g. For GEE, the procedure was the same as that for the RC water extract, except that ethanol was used as the solvent, and an evaporator was used for dehydration.

RC and ginger leaf powder were sequentially extracted thrice with hot water, with each extraction step comprising a 15 min shaking bath at 25 °C followed by 3 min centrifugation (Hettich, universal 16R, USA). The filtrates were pooled and adjusted to 50 mL before freeze-drying (GEA; LYOVAC GT2-S, MD, Hurth, Germany) for 48 h. The final extract was stored and frozen at −20 °C until needed. The RWE and GEE were used for further analyses.

### 2.5. Chromatography Conditions for Polyphenol Analysis

Phenolic compounds in both RC and ginger extracts were quantified using high-performance liquid chromatography (HPLC), following the methodology described by Oonsivilai et al. [10]. Chromatographic separation was performed using a Waters C18 column at 35 °C, maintaining a constant flow rate of 1.0 mL/min. A binary gradient elution system was used with a mobile phase consisting of water/acetic acid (98:2) in reservoir A and acetonitrile in reservoir B. The elution gradient commenced with a 99:1 ratio of A/B, transitioning linearly to 70:30 A/B over a 20 min period. This composition was then held constant for 5 min before returning to the initial 99:1 A/B ratio over 5 min, followed by a 5 min equilibration period. In-line photo diode array (PDA) detection within the 200–500 nm wavelength range facilitated identification and tentative characterization of phenolic compounds. Quantification was achieved by constructing calibration curves using known standards of gallic acid, catechin, caffeic acid, coumaric acid, ferulic acid, sinapic acid, and apigenin at concentrations ranging 5–50 ppm [36].

### 2.6. Cytotoxicity of PM2.5 Exposure A549 and HepG2 Cells

A549 and HepG2 cells were maintained in DMEM supplemented with 10% fetal bovine serum (FBS; HyClone, GA, USA) in a 5% CO_2_ atmosphere at 37 °C. Cells were seeded at a density of 1 × 10^4^ cells/well in DMEM supplemented with 10% FBS in 96-well plates and incubated overnight to allow cell attachment. All subsequent experiments were performed when the cells reached 80–90% confluence, typically after 24 h.

The experiment included two control groups: a reagent control containing only the culture medium, and a solvent control containing 0.1% dimethyl sulfoxide, which was the solvent used to dissolve the extract. Control cells were the test cells that were not exposed to PM2.5. Cells were exposed to various concentrations of PM2.5 (3.125 to 400 µg/mL) in serum-free DMEM for 24 h. Cell viability was assessed using the MTT assay and expressed as a percentage of the control cells [37,38,39].

### 2.7. Cytoprotective Effect of RWE, GEE, and CRGE

To study the cytoprotective effect of all extracts against PM2.5-induced cell toxicity, PM2.5 was used in this study. As a preliminary step, concentrations of PM2.5 which were cytotoxic to cells at a cell viability of approximately 70% were selected for this study. Firstly, A549/HepG2 cells were seeded as described in Section 2.6. Next, the cells were pre-treated for 24 h with RWE, GEE, and CRGE at concentrations that were not cytotoxic to either cell line. They were then exposed to selected PM2.5 for 24 h. Subsequently, the cells were washed with 1X phosphate-buffered saline (PBS). The MTT assay was used to determine the percentage of viable cells.

To investigate the cytoprotective effects of all the extracts on toxins, fine particulate matter (PM2.5) was used. The concentration that revealed cell viability of approximately 70% was used for the cytoprotective study, and cells were pre-treated with RWE, GEE, and CRGE of 50 and 100 µg/mL concentrations for 24 h. Next, cells were washed with 1X PBS and incubated with PM2.5 for 3 h. The MTT assay was used to determine the percentage of cell viability, and the cells exposed to PM2.5 without extracts were used as control [36].

### 2.8. Intracellular ROS Generation

Intracellular ROS production in response to the two toxins was measured using the dichloro-dihydro-fluorescein diacetate (DCFH-DA) assay. HepG2 cells were pre-treated with RWE, GEE, or CRGE for 24 h. After removing the media, cells were exposed to PM2.5 (400 μg/mL) to induce oxidative stress. Following treatment, cells were stained with 20 μM DCFH-DA for 30 min at 37 °C in the dark. Fluorescence intensity was measured using a fluorescence microplate reader (Synergy H1, BioTek, Winooski, VT, USA) at excitation/emission wavelengths of 485/535 nm [36].

### 2.9. Changes in Gene Expression of the Antioxidant Enzymes

A549 and HepG2 cells were used in this study. The cells were seeded at a density of 6 × 10^5^ cells/well in a six-well plate. The cells were incubated with the single and combined extracts, and oxidative stress was induced with PM2.5, as shown in Table 1. All treated cells were washed with PBS and harvested using 0.05% trypsin-ethylenediaminetetraacetic acid (EDTA) [40].

### 2.10. RNA Isolation and cDNA Synthesis

Total RNA was isolated from A549 and HepG2 cells. In brief, cells were lysed using Nucleospin^®^RNA Plus (MACHEREYNAGEL GmbH & Co. KG., Düren, Germany) following the manufacturer’s protocol. The total RNA concentration was determined using an LVis plate and a SPECTROstar NANO system (BMG Labtech GmbH, Ortenberg, Baden-Württemberg, Germany). ReverTra Ace^®^ Quantitative Reverse Transcription-Polymerase Chain Reaction (qRT-PCR) RT Master Mix with gDNA Remover (Toyobo Co., Ltd., Osaka, Japan) was used according to the manufacturer’s instructions for cDNA synthesis. The cDNA samples were frozen at −20 °C for analysis.

### 2.11. qRT-PCR Analysis

Real-time qRT-PCR was performed using SYBR Green (2xqPCRBIO SyGreen Mix Lo-ROX; PCR BIOSYSTEMS) according to the manufacturer’s protocol. cDNA was amplified using a reaction mixture containing 2 µL of cDNA, 2 µL of 2 nM primers, and 5 µL of SYBR Green. PCR conditions were as follows: initial denaturation at 95 °C for 3 min, followed by 40 cycles of denaturation at 95 °C for 30 s, annealing at 60 °C for 30 s, and extension at 72 °C for 1 min. Melting curve analysis was performed to confirm the specificity of the PCR products. Relative gene expression levels were calculated using the 2^−ΔΔCt^ method, with glyceraldehyde-3-phosphate dehydrogenase (GAPDH) as the reference gene. Total RNA was isolated from HepG2 cells. In brief, cells were lysed using Nucleospin^®^RNA Plus (MACHEREYNAGEL GmbH & Co. KG., Düren, Germany) according to the manufacturer’s protocol. The total RNA concentration was determined using an LVis plate and a SPECTROstar NANO system (BMG Labtech GmbH, Ortenberg, Baden-Württemberg, Germany). ReverTra Ace^®^ qRT-PCR Master Mix with gDNA Remover (Toyobo Co., Ltd., Osaka, Japan) was used according to the manufacturer’s instructions for cDNA synthesis. qRT-PCR was performed using SYBR Green (2×qPCRBIO SyGreen Mix Lo-ROX; PCR BIOSYSTEMS, London, UK), according to the manufacturer’s instructions. The data were analyzed using the 2^−ΔΔCt^ expression model and QuantStudioMT Design and Analysis software v.1.52. Relative fold expression of the target gene was normalized to that of GAPDH. Primer sequences for the target genes are listed in Table 2.

### 2.12. Statistical Analysis

Statistical analyses were performed using IBM SPSS Statistics for Windows, version 23.0. Data are presented as the mean ± standard error of three replicates. Student’s *t*-test was used for comparisons between two groups, whereas one-way analysis of variance followed by Duncan’s multiple range test was used for multiple group comparisons. Statistical significance was set at *p* < 0.05.

## 3. Results

### 3.1. Phytochemical Profiling of RWE and GEE

The profile of phenolic and flavonoid compounds in RWE was determined using HPLC analysis (Table 3). The main phytochemical compounds identified in RCWE, in descending order of concentration, include coumaric acid, caffeic acid, proto-catechuic acid, rosmarinic acid, gallic acid, and apigenin. The phytochemical profile of RCWE was based on data from [27], whereas the phytochemical profile of GEE was also investigated and is presented in Table 3.

### 3.2. Cytotoxicity of PM2.5 Exposure A549 and HepG2 Cells

**Figure 1:**
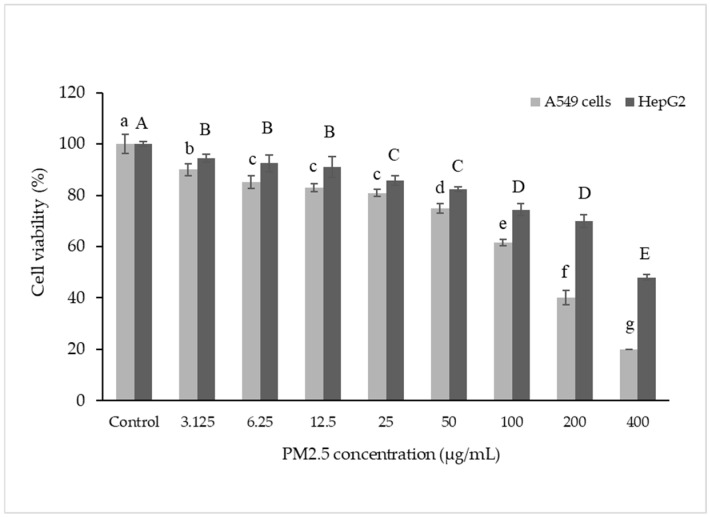
Cell viability in A549 and HepG2 cells. Cells were treated with PM2.5 at 3.125–400 µg/mL for 24 h. Lowercase letters indicate significant differences within A549 cells. Capital letters indicate significant differences within HepG2 Cells. Means sharing the same superscript are not significantly different from each other (*p* < 0.05, two-way ANOVA; Duncan’s method).

The effect of PM2.5 on the survival of A549 and HepG2 cells was investigated. Viability of both cell types was measured after exposure to PM2.5 for 24 h. The results showed that PM2.5 could harm cells by reducing their viability in a dose-dependent manner, indicating that higher PM2.5 concentrations caused greater harm (Figure 1). This finding aligns with a previous study [41,42], which reported a 30% reduction in cell viability at a PM2.5 concentration of 50 µg/mL.

### 3.3. Cell Morphology

**Figure 2:**
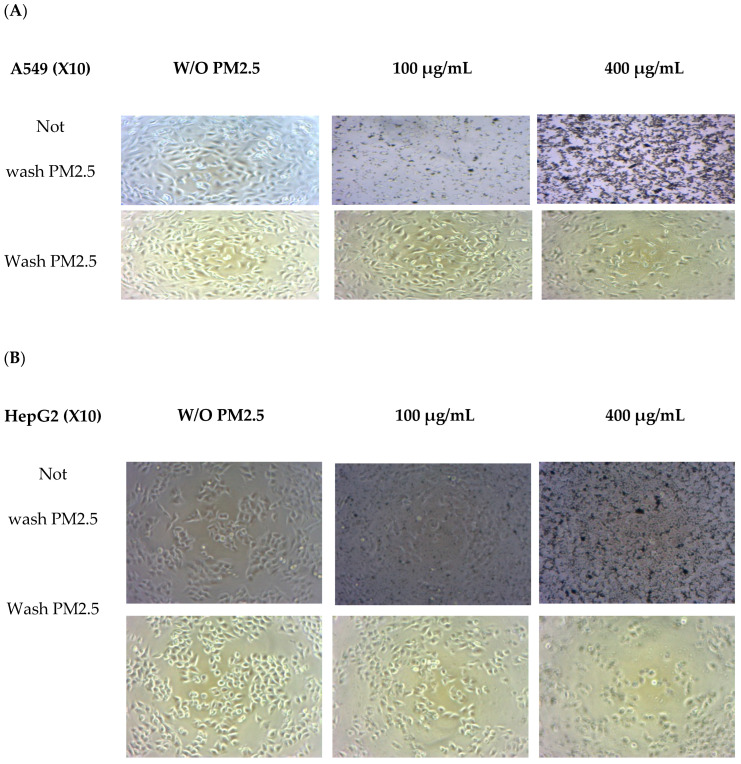
Morphology changes in A549 (**A**) and HepG2 (**B**) cells without PM2.5 exposures and after PM2.5 exposure at 50 and 400 µg/mL for 24 h. A light microscope (×10 magnification) was used.

Morphological changes were observed in both cell lines after exposure to PM2.5 (Figure 2). A light microscope (X10 amplify) was used. Before washing with PM2.5, the number of PM2.5–particles attached to the cells tended to increase with PM2.5 concentration. Morphological observations revealed that cells were affected at higher doses when a high quantity of internalized particles was observed [31]. When washing PM2.5 particles, cell confluence on the plate was reduced at the highest PM2.5 concentration of 400 µg/mL, and overall cell shrinkage was observed. At lower concentrations of PM2.5, the cells adhered to the plate and were related to cell viability.

**Figure 3:**
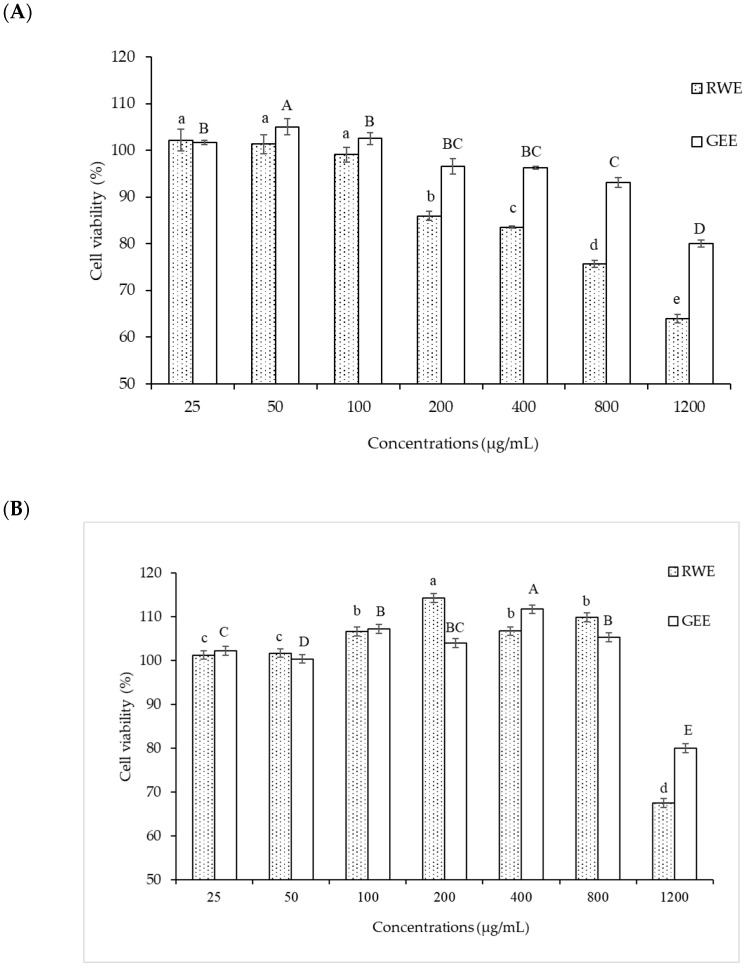
Effect of RWE and GEE at the concentration of 25–1200 µg/mL on cell viability in A549 cells (**A**) and HepG2 cells (**B**) cells. All bars show the mean ± SD of three independent experiments (*n* = 3). Lowercase letters indicate significant differences within RWE. Capital letters indicate significant differences within GEE. Means sharing the same superscript are not significantly different from each other (*p* < 0.05, two-way ANOVA; Duncan’s method).

A549 cells were more sensitive to PM 2.5 exposure than HepG2 cells. As HepG2 cells from the liver remove waste products from the body, they may be resistant to toxic exposure. In the A549 cells model, 50 µg/mL of PM2.5 was used for both cytoprotective effect and intracellular ROS studies by exposure for 24 h. For extract concentration screening (Figure 3), >100 µg/mL of both extracts showed cytotoxicity to A549 cells. Therefore, we selected RWE and GEE for the next study at a concentration of 25 µg/mL in both cell models.

Notably, PM2.5 particles spread extensively in the media before washing, particularly with higher concentrations added. Consequently, washing the cells removed PM2.5 particles that suspended the cells in the medium, leading to decreased cell confluence on the plate. At the highest PM2.5 concentration of 400 µg/mL, overall cell shrinkage was observed.

However, at concentrations of 50 and 400 µg/mL, cells still adhered to the plate, indicating their viability. In summary, A549 cells exposed to PM2.5 exhibited signs of penetration, damage, and destruction.

### 3.4. Cytoprotective Effect of RWE, GEE, and CRGE

**Figure 4:**
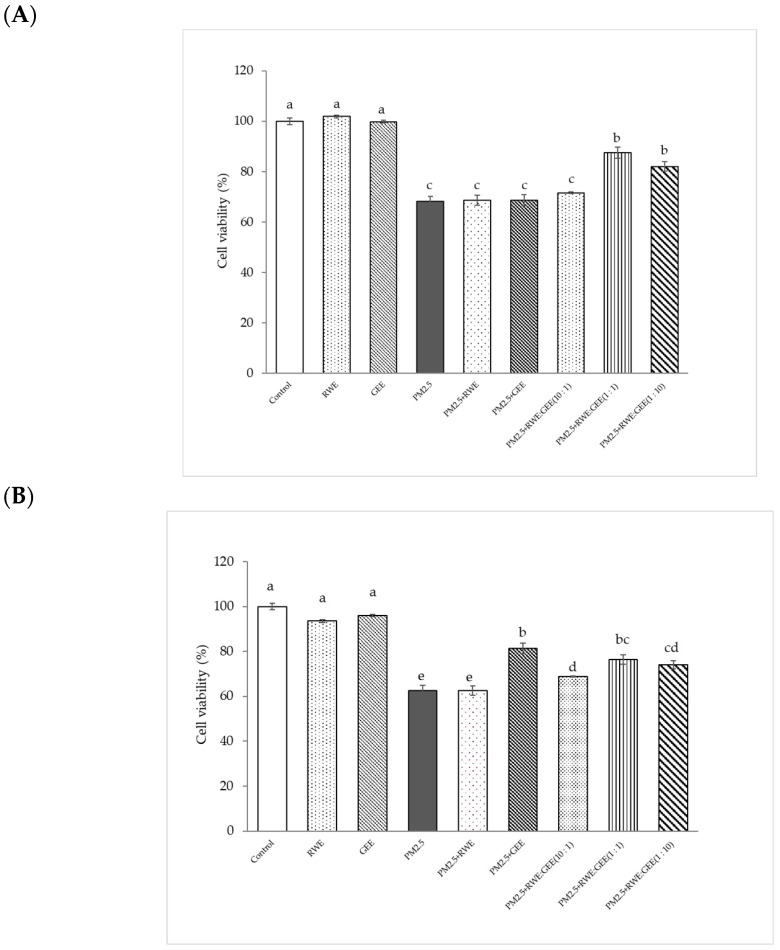
Cytoprotective of extracts from PM2.5-induced cell toxicity in A549 (**A**) and HepG2 (**B**) cells. Cells were treated with extracts at a concentration of 25 µg/mL for 24 h. Next, cells were exposed to PM2.5 at 100 µg/mL for HepG2 cells and at 50 µg/mL for A549 cells for 24 h. Means sharing the same lowercase letters are not significantly different from each other (*p* < 0.05, two-way ANOVA; Duncan’s method).

The cytoprotective effect of extracts attenuated cytotoxicity from PM2.5, and in the A549 cells study, treatment with RWE and GEE did not significantly affect cell viability (%) compared to that seen in the control. Exposure to PM2.5 at 50 µg/mL for 24 h reduced cell viability to 68% (Figure 4A). The major contributor to PM2.5-mediated toxicity is an imbalance between the antioxidant system and oxidation caused by coated particles and/or organic compounds and transition metals [43]. CRGE at ratios of 1:1 and 1:10, followed by exposure to PM2.5, significantly increased cell viability (%) compared to the viability of cells exposed to PM2.5 without extracts. CRGE was more effective in protecting cells from PM2.5 than RWE or GEE alone, even when the single extracts were used at the same concentration. This indicates that the combination of RC and ginger extracts improved cell survival more effectively than the individual extracts.

PM2.5 at 100 µg/mL was used to induce oxidative damage in HepG2 cells. Concentrations of RWE and GEE at 25 µg/mL showed no significant difference when compared to the control without extracts (*p* < 0.05) (Figure 4B). The results showed that GEE and CRGE at three ratios provided greater cell viability protection than RWE and were significantly different from PM2.5-induced cell toxicity without extracts. These results are consistent with the use of RWE: GEE at a ratio of 1:1 to protect HepG2 cells from PM2.5-induced cytotoxicity. Using CRGE at 25 µg/mL could have a cytoprotective effect from PM2.5 exposure for 24 h [44].

### 3.5. Intracellular ROS Scavenging of Extracts from PM2.5

**Figure 5:**
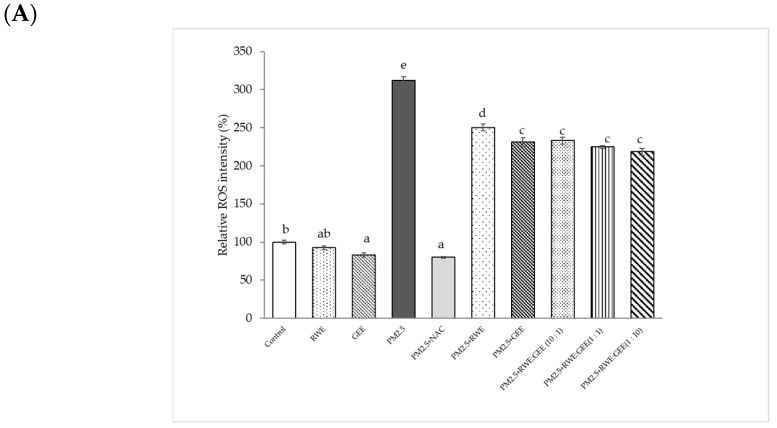

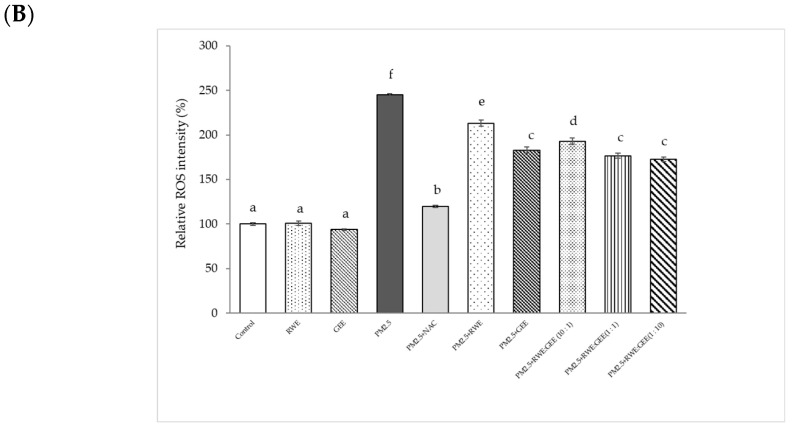
Effect of RWE-, GEE-, and CRGE-attenuated PM2.5-induced intracellular ROS production in A549 (**A**) and HepG2 (**B**). Values are expressed as a percentage of the control. Means sharing the same lowercase letters are not significantly different from each other (*p* < 0.05, two-way ANOVA followed by Duncan’s multiple range test.

To investigate whether the cytoprotective effects of the extracts on the PM2.5-induced toxic response were because of the interruption of oxidative stress, the intracellular ROS-scavenging potential of all extracts was evaluated in the presence of PM2.5-induced oxidative stress (Figure 5). The relative ROS intensity (%) in A549 cells pre-treated with extracts for 24 h and then exposed to PM2.5 was significantly different compared to that in cells exposed to PM2.5 without extracts, reducing ROS intensity from 2-fold to 2.2–2.5-fold relative to the control (Figure 5A). Notably, GEE and CRGE (three ratios) at a concentration of 50 µg/mL significantly decreased relative ROS production compared with that seen with PM2.5 exposure without extracts. However, RWE was not significantly different from that of PM2.5 without extracts (*p* < 0.05). To increase intracellular ROS scavenging of extracts, one may have to increase the concentrations of the extracts by more than 25 µg/mL. Notably, the cell viability of the extracts was not more than 200 µg/mL. PM2.5 showed increased cytotoxicity at 24 h and a significant increase in ROS after 3 h of treatment [31].

Relative ROS intensity (%) in HepG2 cells is shown in Figure 5B. Treatment of HepG2 cells with PM2.5 at a concentration of 25 µg/mL for 24 h increased ROS accumulation approximately 2.5-fold compared to that seen in inactivated HepG2 cells. Treatment with the extracts for 24 h, followed by exposure to PM2.5 significantly reduced ROS intensity by 1.6–2.1-fold compared to that seen in cells exposed to PM2.5 alone. Notably, GEE and CRGE at a concentration of 25 µg/mL significantly decreased relative ROS production compared to RWE pre-treatment groups. These findings suggest that flavonoid compounds present in ginger may play an important role in the protection against oxidative stress. These data demonstrate that CRGE synergistically protects HepG2 cells against PM2.5-induced oxidative stress by improving their ability to scavenge or inhibit ROS production. To increase intracellular ROS, scavenging of extracts may have to increase the concentrations of the extracts by more than 25 µg/mL. Notably, the effect of the studied extracts on cell viability was not more than 200 µg/mL. PM2.5 showed increased cytotoxicity at 24 h and a significant increase in ROS at 3 h of treatment (Figure 5).

### 3.6. Protective Effects of RWE, GEE, and CRGE Against PM2.5-Induced Adverse Effects in Gene Expression of A549 and HepG2 Cells

**Figure 6:**
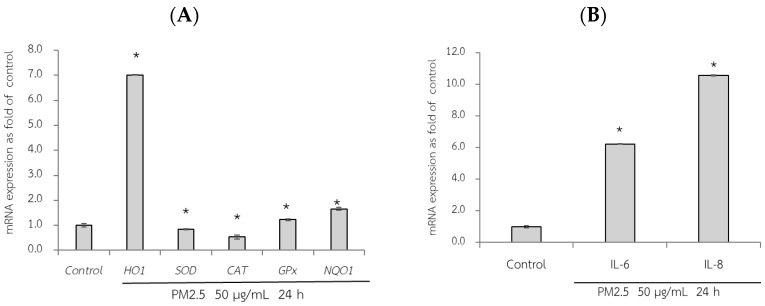
Relative mRNA expressions of antioxidant and phase II detoxification enzymes (HO1, SOD, CAT, GPx, and NQO1) (**A**) and inflammatory cytokines (IL-6, IL8) (**B**) on PM2.5 exposed to A549 cells for 24 h. The control used was cells without exposure to PM2.5. Data are presented as the mean ± SD (*n* = 3). * Above column shows significant differences (*p* < 0.05) from the control without PM2.5 exposure.

**Figure 7:**
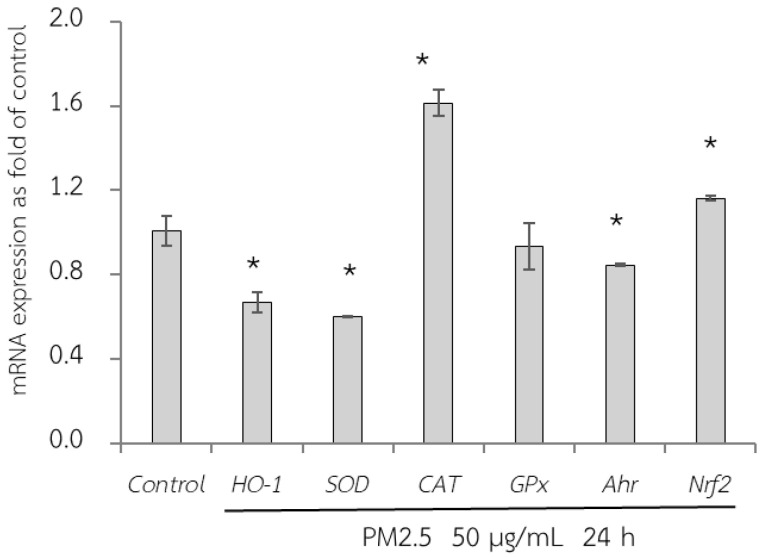
Relative mRNA expressions of antioxidant enzymes (HO-1, SOD, CAT, GPx, Ahr, and Nrf2) on HepG2 cells exposed to PM2.5 for 24 h. The control used was cells without exposure to PM2.5. Data are presented as the mean ± SD (*n* = 3). * Above column shows significant difference from the control without PM2.5 exposure.

First, the adverse effect of PM2.5 on the mRNA expression of A549 and HepG2 cells was analyzed using RT-qPCR. Exposure of A549 cells, which are epithelial lung cells, to PM2.5 represents airway organ contact with PM2.5; therefore, the gene expression of a group of antioxidant enzymes including heme oxygenase-1 [HO-1], SOD, CAT, and GPx, including NQO1 as a phase II detoxification enzyme, and inflammatory markers including IL-6 and IL-8 were investigated. For HepG2 cells, liver cells, considered the major organ for the metabolism and detoxification of xenobiotics, gene expression of antioxidant enzymes (HO-1, SOD, CAT, and GPx) and their regulatory mechanisms (Ahr and Nrf2) were investigated. PM2.5 intake was investigated at the concentration of 50 µg/mL for 24 h, and the maker gene expressions on PM2.5 exposure are shown in Figure 6 and Figure 7.

qRT-PCR analysis revealed the adverse effects of PM2.5, as evidenced by a significant reduction in the expression of SOD and CAT compared to that in the control group (without PM2.5) (*p* < 0.05). Notably, after 24 h of exposure to PM2.5, HO-1 expression increased by approximately seven-fold in A549 cells (Figure 6A). Consequently, PM2.5 exposure resulted in adverse effects on antioxidant and phase II detoxification enzymes, influencing the expression of inflammatory marker genes. However, notably, the sources of the collected PM2.5 may exhibit variations in toxic compositions, duration of exposure to PM2.5, and specific cell models used. These factors can contribute to diverse gene expression results.

Next, qRT-PCR analysis of HepG2 cells exposed to PM2.5 was conducted (Figure 7). In the present study, GPx expression did not change after PM2.5 exposure. However, there was a reduction in HO-1 and SOD expression and an increase in CAT, Ahr, and Nrf2 expression. Nrf2 is a key transcriptional regulator responsible for endogenous antioxidant gene expression and plays a crucial role in defense against oxidative stress. However, although Nrf2 is a regulator of HO-1, our study did not show a concurrent increase in HO-1 expression. This may be caused by the severity of PM2.5 exposure, which downregulated HO-1 expression.

**Figure 8:**
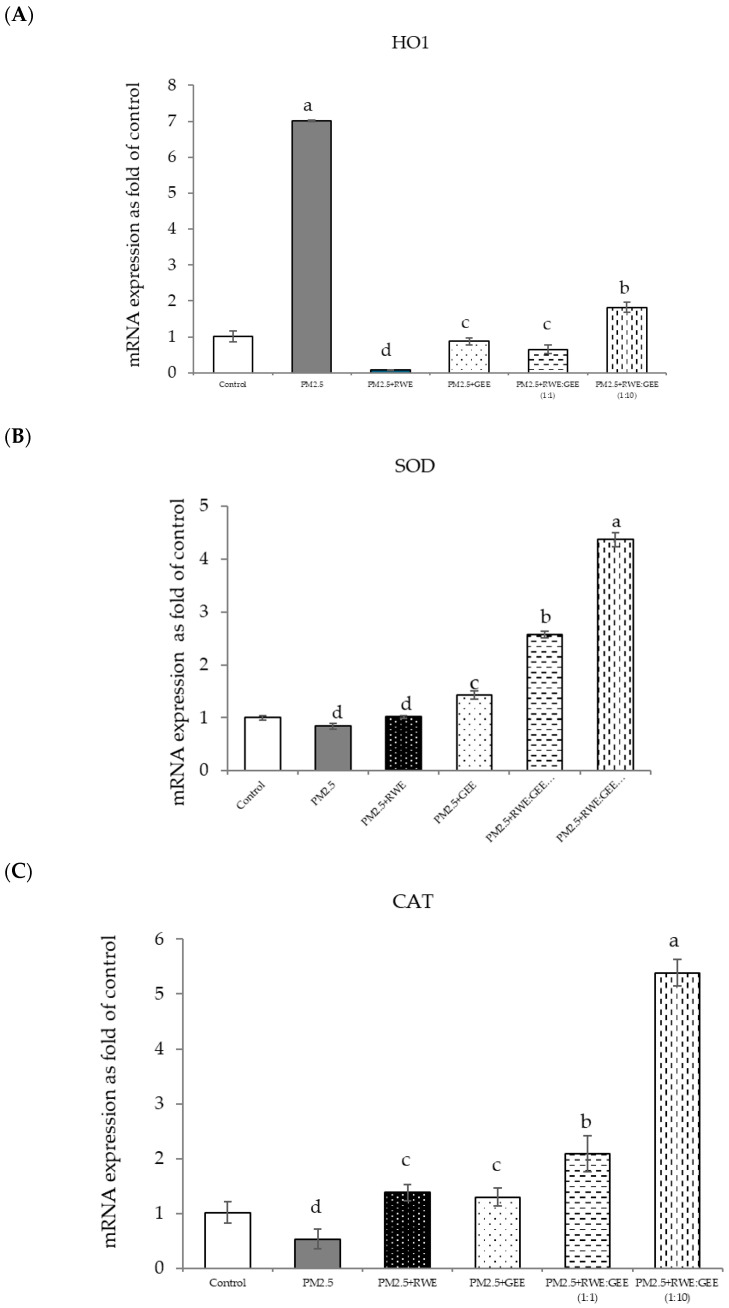

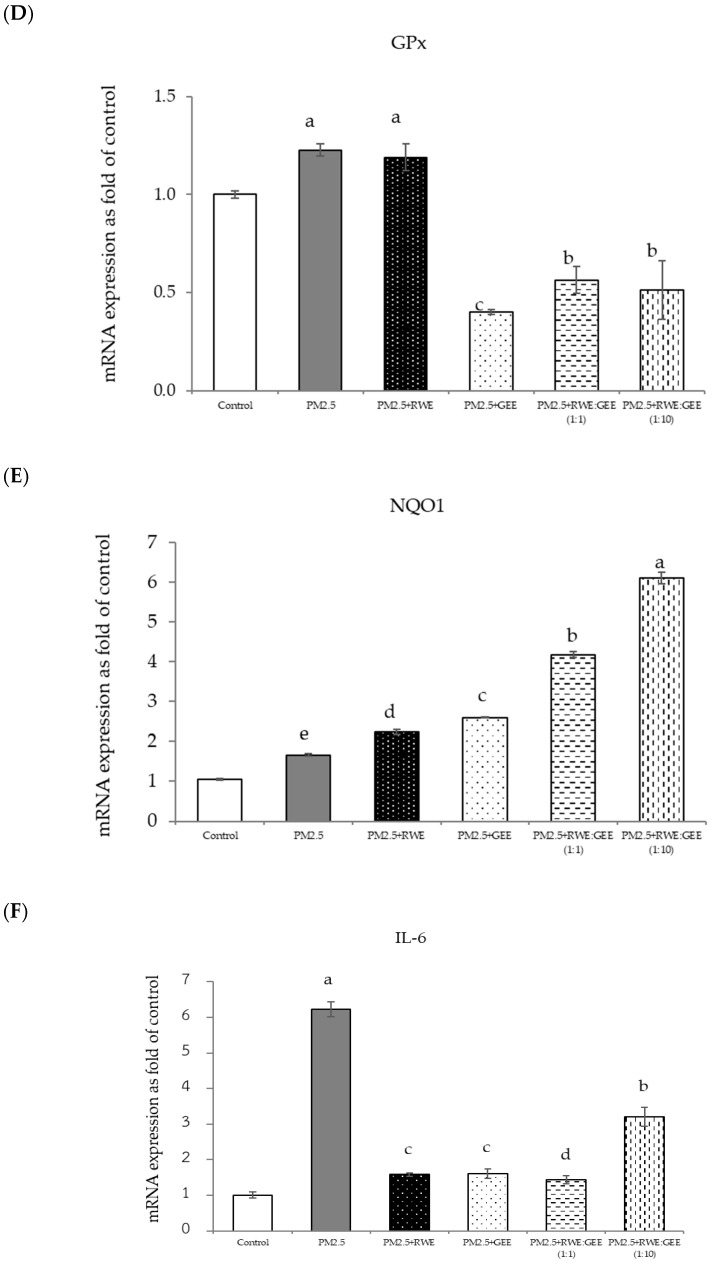

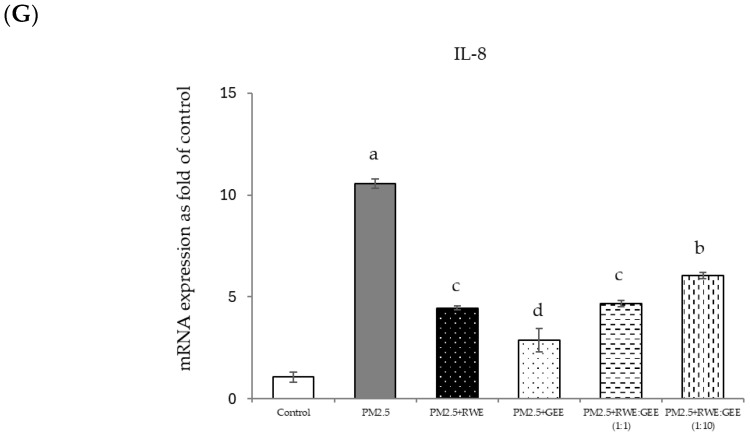
Relative mRNA expressions of antioxidant enzymes and phase II detoxification enzymes and inflammatory cytokines (IL-8) on pre-treatment with single (RWE, GEE) and combined extracts (CRGE; RWE:GEE) on A549 cells exposed to PM2.5. (**A**) HO1, (**B**) SOD, (**C**) CAT, (**D**) GPx, (**E**) NQO1, (**F**) IL-6, (**G**) IL-8. The control was cells without PM2.5 exposure. Data are presented as the mean ± SD (*n* = 3). Means sharing the same lowercase letters are not significantly different from each other (*p* < 0.05, two-way ANOVA; Duncan’s method).

The effects of RWE, GEE, and CRGE on PM2.5-exposed A549 cells and the activities of antioxidant enzymes are presented in Figure 8A–G. The study conditions included a control group, PM2.5 exposure, and four treatment conditions: pre-treatment with extracts (RWE, GEE, RWE: GEE at a ratio of 1:1 *v*/*v*, and RWE: GEE at a ratio of 1:10 *v*/*v*) at a concentration of 50 µg/mL for 24 h, followed by exposure to PM2.5 at 50 µg/mL for an additional 24 h, after which mRNA gene expressions were determined.

**Figure 9:**
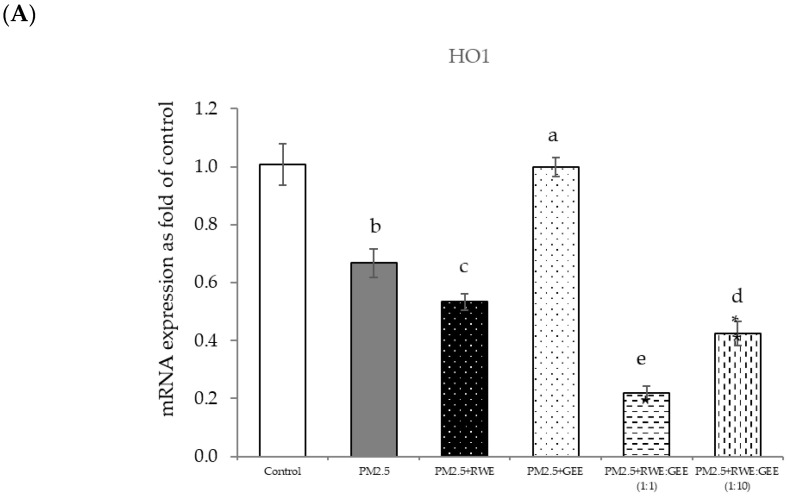

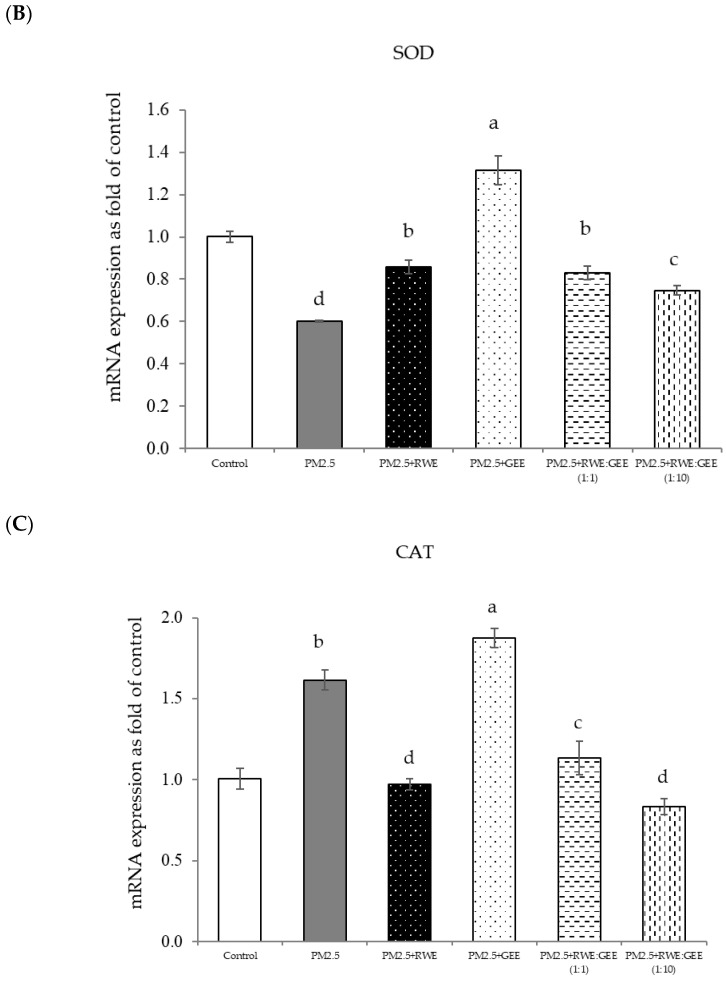

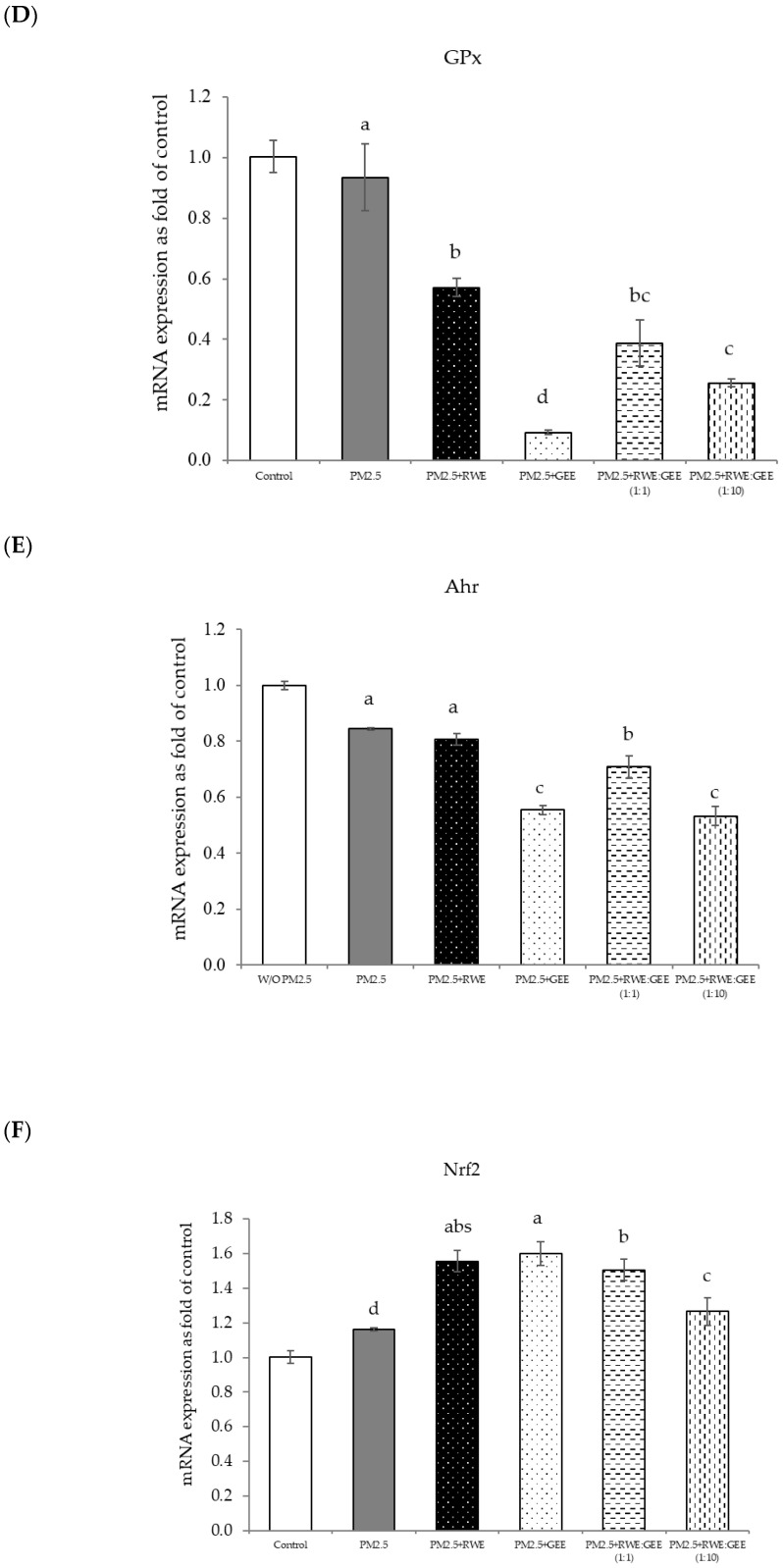
Effect of single and combined extracts on relative mRNA expressions of antioxidant enzymes and antioxidant transcription regulators HepG2 cells exposed to of PM2.5. (**A**) HO1, (**B**) SOD, (**C**) CAT, (**D**) GPx, (**E**) Ahr, (**F**) Nrf2. The control was cells without PM2.5 exposure. Data are presented as the mean ± SD (*n* = 3). Means sharing the same lowercase letters are not significantly different from each other (*p* < 0.05, two-way ANOVA; Duncan’s method).

In the present study, exposure of A549 cells to PM2.5 induced a significant decrease in the concentrations of SOD, CAT, and NQO1. These findings are consistent with the results of studies on the effects of PM2.5 on A549 cells, and these effects were reversed by pre-treatment with the single and combined extracts for 24 h. In the qRT-PCR analysis, RWE and GEE were individually tested in HepG2 cells (Figure 9). RWE significantly upregulated the expression of SOD and CAT 2.5- and 3.0-fold, respectively. In addition, the activities of CAT and GPx were elevated compared to their control levels. Previous studies have shown that certain plant extracts, including trumpet extract and caffeic acid, can enhance the activity of antioxidant enzymes, such as CAT. Although RWE primarily affected SOD and CAT, GEE influenced the expression of OH-1. These varying responses to the different extracts indicate diverse mechanisms of action. CRGE emerged as the most potent extract, upregulating the expression of CAT and SOD, thereby promoting their enzymatic activity.

## 4. Discussion

The phytochemicals identified in GEE in this study included caffeic acid, protocatechuic acid, rosmarinic acid, and apigenin, which have previously been reported [9,10]. CRGE attenuates H_2_O_2_-induced cytotoxicity in HepG_2_ cells. In this study, H_2_O_2_ was used to induce oxidative damage as it is a stable source of free radicals and is frequently used in the development of in vitro oxidative stress models. H_2_O_2_ is one of the most important ROS involved in redox control of biological activity. However, at excessive intracellular concentrations, ROS and free radicals severely damage the cells and tissues, resulting in decreased mitochondrial membrane potential, protein damage, and DNA fragmentation [45]. This led us to study the protective effects of the phenolic compounds in RC against oxidative stress. PM2.5 generates oxidative stress by inducing the overproduction of ROS. Thus, we investigated the effect of CRGE on PM2.5-induced intracellular ROS generation in HepG_2_ cells. Preliminarily, exposure of HepG2 cells to PM2.5 for 3 h increased ROS intensity more than exposure for 24 h. In addition, ROS production was observed at early exposure times, indicating that organic and inorganic water-soluble components of PM2.5 may cause this early response [31]. A 3 h exposure of A549 cells to PM2.5 significantly augmented ROS accumulation. Compared to inactivated control cells, ROS levels demonstrated an approximately three-fold increase. Moreover, upon exposure to PM2.5 at a concentration of 25 µg/mL, ROS intensity exhibited a substantial 190% elevation. However, the solubility of PM2.5, such as organic solvent-extractable or water-soluble fractions, affects ROS generation and cytotoxicity [41], increasing ROS production by PM2.5 exposure as heavy metals can generate ROS at low levels [42].

The combined extracts at ratios of 1:1 and 1:10 demonstrated a greater increase in cell viability than did RWE or GEE alone, suggesting a synergistic interaction between the components of the combined extract. Moreover, the cytoprotective effect of CRGE was observed at a concentration of 25 µg/mL, whereas individual extracts at the same concentration did not show a significant difference from the PM2.5-exposed control. These results suggest that CRGE protects cells from PM2.5-induced oxidative damage by mitigating the cytotoxic effects of PM2.5.

The gene expression of various antioxidant enzymes was inconsistent when cells were exposed to PM2.5, likely owing to the complex interplay of multiple mechanisms. Previous studies have demonstrated that exposure to PM2.5 can activate Nrf2 signaling pathway, leading to the upregulation of antioxidant genes, such as HO-1 and NQO1, and subsequently inducing oxidative stress [43,46]. Nrf2, a key transcription factor, binds to antioxidant response elements (AREs) to regulate the expression of genes involved in cellular defense against oxidative stress. The reduced expression of SOD and CAT observed in this study may be associated with decreased SOD enzyme activity during PM2.5 exposure [32]. SOD is an antioxidant enzyme that scavenges free radicals and converts superoxide radicals into hydrogen peroxide, thereby providing cytoprotection against damage caused by toxic oxygen free radicals [47]. The findings of this study align with previous research indicating that PM2.5 exposure can diminish the activity of antioxidant enzymes such as SOD and CAT in A549 cells [48]. These results suggest that PM2.5 induces the generation of ROS, which can directly interact with and impair the function of antioxidant enzymes, such as SOD, GPx, and CAT, leading to decreased enzymatic activity. However, it is noteworthy that GPx was upregulated upon exposure to PM2.5, as it primarily acts as a hydrogen peroxide scavenger by catalyzing glutathione oxidation and using hydroperoxide as a substrate. In conclusion, PM2.5 induces oxidative stress-related damage in A549 cells by disrupting antioxidant gene expression and compromising the antioxidant defense system.

In terms of inflammatory cytokine expression, we observed the upregulation of inflammatory response markers, specifically IL-6 and IL-8 (Figure 6B), indicating an inflammatory response. These findings align with previous studies [49] indicating that PM2.5 exposure can trigger inflammatory cytokine production in A549 cells, potentially via the activation of the TLR4/NF-κB/COX-2 signaling pathway. This pathway was also documented in a study involving RAW264.7 macrophages exposed to PM2.5 [50]. These pro-inflammatory effects can be attributed to the toxic components of PM2.5, such as PAHs and metals (e.g., arsenic, lead, iron, and aluminum). These constituents have been identified as major contributors to the inflammatory response [51]. Nrf2 is a key transcription factor that is activated during oxidative stress. Upon dissociation from Keap1, Nrf2 translocates to the nucleus, where it initiates the transcription of genes encoding protective phase II enzymes. This transcriptional activation helps restore cellular redox homeostasis [52]. This suggests that PM2.5 exposure induces the upregulation of Nrf2, a master regulator of the cellular antioxidant response. Activated Nrf2 binds to AREs, stimulating the expression of phase II antioxidant enzymes, including heme oxygenase-1 (HO-1) [47].

Numerous studies have consistently demonstrated the essential role of the Nrf2-antioxidant response pathway in combating oxidative stress induced by PM2.5. Nrf2 plays a crucial role in bolstering the cellular defense system by stimulating antioxidant genes. It protects against inflammation and detoxifies environmental electrophiles such as aromatic hydrocarbon quinones, crotonaldehyde, acrylamide, methylmercury, and cadmium [49]. Particulate matter, organic chemicals, and transition metals can disrupt the balance between oxidation and antioxidant systems, leading to PM2.5-induced toxicity [43]. This leads to the overproduction of ROS following oxidative stress, which subsequently depletes antioxidants, resulting in the breakdown of endogenous antioxidant defense mechanisms, ultimately failing to protect cells from damage induced by oxidative stress. To further assess how the cellular antioxidant activities of the extracts influenced H_2_O_2_-activated ROS production and cell cytotoxicity, the upregulated mRNA gene expression of antioxidant enzymes was investigated. As shown in Figure 4, RWE and CRGE significantly upregulated the transcription of HO-1, SOD, CAT, and NQO1 transcription in H_2_O_2_–activated HepG2 cells. This finding aligns with previous research demonstrating that RC leaf extracts can upregulate antioxidant enzymes such as CAT and GPx. HO-1, an inducible stress response protein, is primarily regulated by Nrf2. Nrf2 plays a crucial role in the cellular antioxidant defense by activating the expression of phase II antioxidant enzymes, including HO-1 and NQO1. In response to oxidative stress, Nrf2 dissociates from Keap1, translocates to the nucleus, and binds to AREs to upregulate the expression of target genes. The observed increase in Keap1 expression coupled with a decrease in Nrf2 suggests a compensatory mechanism to protect cells from oxidative damage [47].

This can be attributed to the composition of RC, notably rosmarinic acid, which exhibits direct scavenging capability and provides molecular protection. Evidence from Fetoni et al. [53] suggests that RA increases SOD and HO-1 levels, indicating the activation of the cell stress response in an in vivo model, and that RA potentiates Nrf2/HO-1 signaling. Furthermore, gallic acid in RWE activated the Nrf2/HO-1 pathway and reduced oxidative stress [54,55,56].

Focusing on the combined extracts at two different ratios, these CRGE formulations surprisingly upregulated the expression of antioxidant genes, including HO-1, SOD, CAT, GPx, and NQO1, which serve as phase II antioxidant enzymes (Figure 4 and Figure 8A–C,E). Moreover, an RWE: GEE ratio of 1:10 resulted in the highest level of expression, which may be attributed to the interaction between the extracts in the correct proportions. The increase in antioxidant enzyme activity has been supported by several studies. For example, phenolic hydroxyl groups in tea polyphenols have been shown to interact with phenolic substances in kelp, forming stable chemical complexes [45]. However, these studies focused on specific plants of interest and may not provide a comprehensive view of the entire mechanism that may occur. Notably, HO-1 and GPx were downregulated by all the extracts.

This study revealed that the protective effect of the single and combined extracts against inflammation was achieved by reducing the expression of the inflammatory cytokines IL-6 and IL-8. Benzo[a]pyrene, a major component of PM2.5, increases IL-6 and IL-8 levels by activating the EGFR-ERK1/2 signaling pathway [47]. Zingerone, an active ingredient in ginger, activates the AMRK/Nrf2/HO-1 signaling pathway [49]. The results of this study showed that CRGE represents a valuable therapeutic target for mitigating oxidative damage to lung cells caused by PM2.5. However, further investigation of Nrf2 protein expression levels and nuclear translocation is warranted to clarify the underlying mechanisms.

## 5. Conclusions

This study investigated the antioxidant and cytoprotective potential of RWE and GEE, both individually and in combination. This combination in a 5:1 ratio (CRGE) significantly enhanced antioxidant activity and cytoprotective effects against oxidative stress, surpassing individual extracts and commercial standards. The synergistic mechanism involves both free radical scavenging and the modulation of gene expression related to antioxidant enzymes. To explore the potential of CRGE in mitigating oxidative stress induced by environmental pollutants, PM2.5 was used as a model toxin. Exposure to PM2.5 downregulated CAT expression compared to that in the control group, indicating increased oxidative stress. However, no significant changes were observed in the expression of SOD or GPx. The excessive ROS generated by PM2.5 leads to cellular oxidative damage and further downregulation of antioxidant enzyme gene expression. CRGE effectively reduced ROS production induced by PM2.5 exposure, providing cytoprotection to HepG2 and A549 cells. The combined extract mitigated PM2.5-induced cytotoxicity and scavenged intracellular ROS. In addition, CRGE upregulated the expression of antioxidant enzymes, including OH-1, SOD, and CAT, in response to PM2.5, thereby enhancing the antioxidant capacity of cells.

Although these results are encouraging, additional research is necessary to fully understand the mechanisms underlying the synergistic effects of CRGE and its potential applications. The limitations include the reliance on in vitro models and the heterogeneity of PM2.5. Future studies should focus on measuring antioxidant enzyme activities, conducting in vivo experiments, and utilizing bioinformatics analysis to identify key target genes and signaling pathways involved in the protective effects of CRGE.

## Figures and Tables

**Table 1:** Testing conditions on gene expression.

Treatments	Pre-Treatments	Activation
Control	Media (24 h)	Media (24 h)
PM2.5 (Negative control)	Media (24 h)	PM2.5 (24 h)
RWE	RWE 50 µg/mL (24 h)	Media (24 h)
GEE	GEE 50 µg/mL (24 h)	Media (24 h)
RWE + PM2.5	RWE 50 µg/mL (24 h)	PM2.5 50 µg/mL (24 h)
GEE + PM2.5	GEE 50 µg/mL (24 h)	PM2.5 50 µg/mL (24 h)
RWE: GEE (1:1) + PM2.5	RWE: GEE (1:1) 50 µg/mL (24 h)	PM2.5 50 µg/mL (24 h)
RWE: GEE (1:10) + PM2.5	RWE: GEE (1:10) 50 µg/mL (24 h)	PM2.5 50 µg/mL (24 h)

**Table 2:** Target gene primer sequences used to investigate the cytoprotective potential of *Thunbergia laurifolia* Lindl. combined with ginger extracts against the adverse effects of PM2.5.

Target Gene	Forward Primer Sequence (5′ to 3′)	Reverse Primer Sequence (3′ to 5′)
HO-1	ATGGCCTCCCTGTACCACATC	TGTTGCGCTCAATCTCCTCCT
SOD1	GCAGGTCCTCACTTTAATCCTCT	ATCGGCCACACCATCTTTGT
CAT	TGAAGATGCGGCGAGACTTT	TGGATGTAAAAAGTCCAGGAGGG
NQO1	GGATTGGACCGAGCTGGAA	AATTGCAGTGAAGATGAAGGCAAC
GAPDH	TCCAAAATCAAGTGGGGCGA	TGATGACCCTTTTGGCTCCC

**Table 3:** Phytochemical profiles of Rang Chuet and ginger extracts analyzed using HPLC.

Photochemical		RWE (µg/g of Extract)	GEE (µg/g of Extract)
Phenolic compounds	Gallic acid	128 ± 11	ND
	Proto-catechuic acid	418 ± 14	ND
	Caffeic acid	919 ± 20	125 ± 0.58
	Coumaric acid	1025 ± 49	35 ± 0.81
	Ferulic acid	ND	72 ± 0.89
	Sinapic acid	ND	ND
	Rosmarinic acid	374 ± 14	ND
Flavonoids	Quercetin	ND	ND
	Apigenin	107 ± 2.9	ND
Total phenolic compounds and flavonoids		2976 ± 19	232 ± 0.76

ND: No data available.

## Data Availability

The original contributions presented in this study are included in the article and Supplementary Materials. Further inquiries can be directed to the corresponding authors.

## Supplementary Materials

The following supporting information can be downloaded at: https://www.mdpi.com/article/10.3390/foods14030517/s1, Figure S1. Particle size distribution for SRM 2786 after 1 h. The solid line represents the volume in percentage. Table S1. Analyze composition in SRM 2786. Table S2. Inorganic Constituents in SRM 2786. Table S3. Ginger root information.
