# Supplementary material for: Synergistic Antioxidant and Cytoprotective Effects of Thunbergia laurifolia Lindl and Zingiber officinale Extracts Against PM2.5-Induced Oxidative Stress in A549 and HepG2 Cells
Source: Foods. 2025 Feb 5;14(3):517. doi: 10.3390/foods14030517 (PMC11817398; doi:10.3390/foods14030517)
Supplement: Supplementary file 1 [file foods-14-00517-s001.zip › foods-3347160-supplementary.pdf]

Supplementary documents

Information about Standard Reference Material® 2786 Fine Atmospheric Particulate Matter

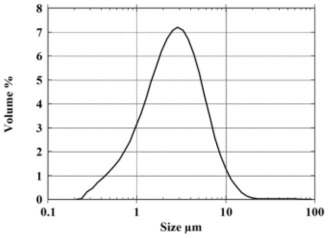

Figure S1. Particle size distribution for SRM 2786 after 1 h. The solid line represents the volume in percentage.

Table S1. Analyze composition in SRM 2786

composition	*Mass Fraction (μg/kg)*
Inorganic constituents	167,794.9
Nitro-substituted PAHs (nitro-PAHs)	1,411.9
Sugars	592.9
Hexabromocyclododecane (HBCD) isomers	561.0
Polybrominated diphenyl ether (PBDE) congeners	10.5
Polycyclic aromatic hydrocarbons (PAHs)	7.9
Dibenzofuran (PCDF) congeners	7.3
Polychlorinated dibenzo-p-dioxin (PCDD)	6.0

Table S2. Inorganic Constituents in SRM 2786

Inorganic Constituents	Mass Fraction (μg/kg)
Calcium	73500 ± 1900
Iron	48900 ± 2400
Magnesium	8300
Chlorine	17390 ± 440
Sodium	14920 ± 370
Titanium	2460 ± 140
Zinc	1793 ± 88
Nickel	243 ± 4
Antimony	192.1 ± 9.4
Arsenic	36.7 ± 1.1
Lanthanum	20.72 ± 0.68
Cobalt	19.55 ± 0.96
Scandium	7.2
Thorium	5.8
Cesium	4.01 ± 0.22
Samarium	2.840 ± 0.090

Table S3. Ginger root information

**Major component(s)**	**Manufacturer**	**Product name**	**Matrices**	**Lebel claim**	**Estimated level (w/w purity)**
**Ginger**	Now Foods	Ginger Root extracts, 250 mg	Capsule	12.5 mg gingerols/capsules (498.02 mg/capsule)	2.51% total constituents
